# The association study between changes in HbA1C with rs2250486 and rs67238751 genetic variants for *SLC47A1* in newly diagnosed Iranian patients with type 2 diabetes mellitus: 6 months follow‐up study

**DOI:** 10.1002/edm2.410

**Published:** 2023-02-14

**Authors:** Armina Semnani, Faranak Kazerouni, Saeid Kalbasi, Seyedeh Zahra Shahrokhi, Ali Rahimipour

**Affiliations:** ^1^ Department of Clinical Biochemistry Shahid Beheshti University of Medical Sciences Tehran Iran; ^2^ Department of Medical Lab Sciences, School of Allied Medical Sciences Shahid Beheshti University of Medical Sciences Tehran Iran; ^3^ Department of Clinical Endocrinology, School of Medicine Shahid Beheshti University of Medical Sciences Tehran Iran; ^4^ Department of Biochemistry, School of Medicine AJA University of Medical Sciences Tehran Iran

**Keywords:** HbA1C, metformin, SLC47A1, type 2 diabetes

## Abstract

**Objectives:**

One of the most well‐known oral medications for the treatment of T2DM is metformin. Variants have been found in studies to be useful in detecting new genes connected to T2DM aetiology and affecting metformin's mechanism of action. In this research, we aimed to study two variations of the *SLC47A1* gene; rs2250486 and rs67238751, in T2DM patients who had been taking metformin for the first 6 months after the diagnosis in the Iranian population for the first time.

**Design and Methods:**

A total of 200 individuals were recruited for the study. According to their glycosylated haemoglobin (HbA1c) levels, the patients were divided into two groups: responders (HbA1c levels were reduced by at least 1% after 6 months of metformin treatment.) and non‐responders. DNA was extracted from whole blood and genotyped by Tetra ARMS PCR. High‐performance liquid chromatography (HPLC) was used to measure HbA1c levels at the start of the treatment and again 6 months later.

**Results:**

rs2250486 variant in the dominant model reduces the HbA1C levels after 6 months of metformin treatment. In fact, when compared to the T/C + C/C genotypes, the T/T genotype improves HbA1C levels (*p*‐value = .014). Furthermore, in the allelic model, the T allele improves HbA1C levels in comparison to the C allele (*p*‐value = .008). After 6 months of metformin treatment, serum levels of HbA1C in responders were reduced significantly in both groups (T/T and T/C + C/C), (*p*‐value = <.0001). However, the rs67238751 variant did not reveal a meaningful relationship with lower HbA1C levels in any of the models.

**Conclusions:**

This study found that the rs2250486 variant could be associated with reducing HbA1C levels while the rs67238751 variant, had no relationship.

## Graphical abstract

In this research, we aimed to study two variations of the SLC47A1 gene; rs2250486 and rs67238751, in T2DM patients who had been taking metformin for the first 6 months after the diagnosis in the Iranian population for the first time. This study found that the rs2250486 variant could be associated with HbA1C changes while the rs67238751 variant, had no relationship.

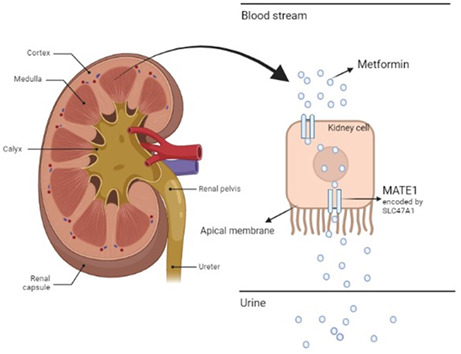

## 1 INTRODUCTION

**FIGURE 1:**
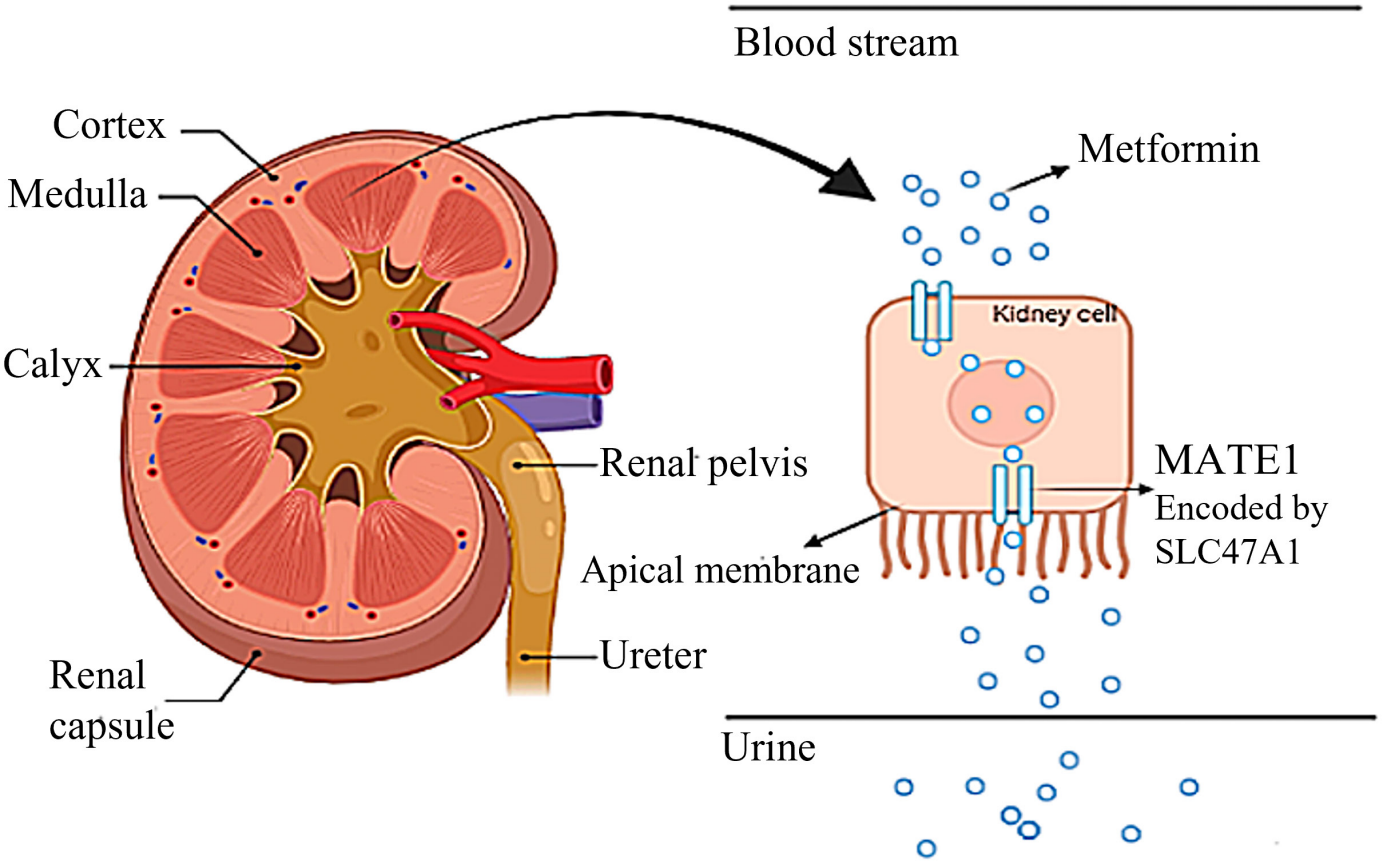
Schematic figure of how metformin is secreted into urine by MATE1.

Type 2 diabetes mellitus (T2DM) is one of the most common metabolic disorders worldwide. Deficiency in insulin release by pancreatic beta cells and decreased tissue sensitivity to insulin are the two main causes of the disease. The insulin synthesis and molecular secretion mechanisms, as well as the insulin response in target tissues, must be ideally co‐ordinated. As a result, a defect in the involved mechanisms can lead to a metabolic imbalance, which can contribute to the pathogenesis of T2DM.^1^, ^2^ Having a genetic background plays an important role in T2DM. The polygenic nature of the disease has been discovered in studies during the last decade. The majority of these genes have the ability to influence insulin secretion and its activity.^3^ One of the most frequent medications used to treat T2DM is metformin, which is made from biguanide. Metformin can be used single or in combination with other medications like insulin to treat diabetes.^4^ Metformin is actively transported through organic cation transporters (OCTs) in enterocytes, liver cells, and renal epithelial cells. Multidrug and toxin extrusion transporter 1 (MATE1), encoded by *SLC47A1* transports metformin from liver to the bloodstream. Finally, metformin is secreted into the urine via MATE1 and MATE2 (encoded by *SLC47A2*)^5^, ^6^ (Figure 1). On a single‐medication treatment with metformin, over 35% of patients with T2DM fail to regulate their serum blood glucose concentrations.^7^ The relationship between genetic variations of metformin transporters and treatment results has been discovered in a number of studies.^8^ Genetic variations can affect gene expression in a variety of ways, resulting in changes in treatment responsiveness.^9^ Expression quantitative trait loci (eQTLs) are defined as genetic variations that are linked to gene expression and are available at the genotype‐tissue expression (GTex) portal.^10^ Variations in the *SLC47A1* gene, which encodes one of the metformin kidney transporters, might have a significant impact on metformin reabsorption via MATE1. However, only a few contradictory studies on the effect of the *SLC47A1* gene on metformin efficiency have been conducted around the world.^11^ The current study focuses on two intronic variants in the *SLC47A1* gene, rs2250486 (T > C) and rs67238751 (C > T), which might have an impact on metformin responsiveness and reducing HbA1C levels in T2DM patients in the Iranian population for the first time.

This study evaluated the effects of these variants on HbA1c levels and other biochemical and anthropometric parameters like systolic and diastolic blood pressure (SBP, DBP), lipid profile, fast blood sugar (FBS) and body mass index (BMI) before and after metformin treatment in both responder and non‐responder groups. It also looked at the effect of dominant, co‐dominant and recessive models in the genotypic groups with the effect of metformin treatment.

## 2 MATERIALS AND METHODS

### 2.1 Study population

This research is a case–control study. Prior to participating in this research, all individuals gave their informed consent in accordance with the Helsinki Declaration's principles, and the protocol was approved by the Shahid Beheshti University of Medical Sciences' Local Ethics Committee (reference no IR.SBMU.MSP.REC.1399.119). According to similar samples in our last study,^12^ 200 patients over the age of 45, both men and women, were referred to the TABAN Diabetes Clinic and newly diagnosed with T2DM based on world health organization (WHO) recommendation.^13^ Patients with liver disorders such as hepatitis, infectious and inflammatory diseases, kidney and autoimmune diseases, and pregnant women were excluded from the trial, as were those who received insulin or other oral antidiabetic medications. The number of people in the group who responded to treatment was 102 (The reduction in HbA1C levels was more than 1% after 6 months of monotherapy with metformin). Whereas the number of people who did not respond to metformin treatment was equal to 98.

### 2.2 Blood sampling

A questionnaire was used to collect information such as age, sex and history of endocrine, hepatic, infectious, and inflammatory disorders, as well as a history of blood‐sugar‐lowering medications and the length of diabetes. Biochemical and anthropometric parameters were analysed at baseline and after 6 months of monotherapy. Blood sampling was performed after 10–12 h of fasting. FBS, triglyceride (TG), total cholesterol (TC), low‐density lipoprotein cholesterol (LDL‐C) and high‐density lipoprotein cholesterol (HDL‐C) were measured by the HITACHI auto‐analyser. sdLDL was calculated through the TG/HDL formula. HbA1c levels were analysed by the HPLC. Individuals' weight and height were measured using standard procedures, and BMI was calculated using the standard formula (kg/m^2^). Blood pressure was measured in a sitting position and after 15 min of rest using a standard blood pressure monitor.

### 2.3 Treatment protocol

We enrolled 200 Iranian patients who were newly diagnosed with T2DM, and all patients were only treated with metformin for the first 6 months after the diagnosis. An identical dose (1000 mg/day) was given to all of the participants. All patients were instructed to follow a low‐carb diet under the guidance of a dietician after the initial diagnosis and the beginning of treatment, and to walk on average 3 days per week.

### 2.4 Genotyping

Three millilitre of patients' blood was stored at −80°C in vials containing ethylenediaminetetraacetic acid (EDTA) until the day of DNA extraction. The salting‐out approach, which is a quick and cost–benefit method for DNA extraction, was applied in this work. The concentration of extracted DNA, as well as the purity of the samples, were determined using a Nano‐drop spectrophotometer. For each variant, Tetra ARMS PCR was used for genotyping. The thermocycler setup programs for the variants are shown in Table 1. After the PCR, the products were evaluated using a 2% agarose gel electrophoresis. For primer design, the primer1 program was used. For the rs67238751 variant, the designed primers were forward outer 5′‐AGAACTGACGAGAAGAGGAAACATC‐3′, reverse outer 5′‐GGAAAAAGTTGTGGAAAGTAGTCTC‐3′, forward inner (C allele) 5′‐TACCAAACTTAATCACAGGGGGC‐3′, reverse inner (T allele) 5′‐GTTAGAGGTGCCGTTTCGCA‐3′ and for rs2250486 variant were forward outer 5′‐ TATAGATGACTGAAAGGTCCCCAGTTCC‐3′, reverse outer 5′‐GAAACAACACAACTGACCACTGACTAAG‐3′, forward inner (T allele) 5′‐GATTCAGATTGTGCAAATGTGGTGAT‐3′, reverse inner (C allele) 5′‐GGGATTACTCAACTGACTGAAGAAAAACG‐3′.

**TABLE 1:** Thermocycler settings to detect rs2250486 and rs67238751 variants.

Stage name	Duration	Number of repetitions	rs2250486	rs67238751
			Temperature (°C)	
Initial denaturation	5′	Cycle 1	95	95
Denaturation	30′′	Cycle 35	94	94
Annealing	30′′		57	64
Extension	30′′		72	72
Final extension	10′′	Cycle 1	72	72

### 2.5 In silico analysis

The PharmGKB (https://www.pharmgkb.org/) database was used to find metformin‐related genes, and after selecting the *SLC47A1* gene based on its important role in the liver and renal tissues in the publications, the variations of this gene were taken from the GTex portal, which is based on eQTL. Although ancestral allele, Genetic population and flanking sequence for selected variants were discovered using Ensemble database. These single nucleotide variations are intronic. The position areas for rs2250486 and rs67238751 are chr17:19573275 and chr17:19562914 respectively. Using HaploReg online tool (https://pubs.broadinstitute.org/mammals/haploreg/haploreg.php) also showed that rs67238751 changes the binding affinities for the transcription factors *CAC*‐binding‐protein*, CACD, CTCFL, E2F, Irf, MAZ, MYC, Pax‐4, SP1, SP2, SRF, SP4*. The regulatory motif for *Irf* contains rs2250486. Thus, both variations might change how the host's genes are expressed.

### 2.6 Statistical analysis

PGA software was used to calculate the sample size.^14^ In this study, the abundance of alleles and genotypes and Hardy–Weinberg equilibrium were investigated using SNPStats software (https://www.snpstats.net/). Patients' data were analysed using SPSS software (version 25). Descriptive statistics in the present study are expressed as the average ± SD. The Shapiro–Wilk test was used to determine the normality of the data distribution. Also, a 2‐way ANOVA test was used to compare parametric variables.

## 3 RESULTS

### 3.1 Patients' characteristics

The study included 200 patients, 97 men and 103 women, and found that 102 patients responded to metformin treatment, and 98 patients did not. There were 48 women and 54 men among the responders. There were 57 women and 41 men among the non‐responders. The average age ranged from 55.84 ± 4.8 in the responder group and 56.99 ± 4.85 in the non‐responder group. The difference between these two groups was not significant (*p*‐value = .9). The Mann–Whitney *U* test was utilized due to the anomalous age distribution in both groups. Table 3 shows the genotypes and allele distributions of the studied variations. The two variations have a significant correlation (*D*′ = 0.307, *r* ^2^ = 0.073) according to online SNPSTATs software.

### 3.2 Genotypic frequencies

#### 3.2.1 rs2250486 (T > C)

According to data analysis, the distribution of genotypes in the two groups of responders and non‐responders is in Hardy–Weinberg equilibrium (*p*‐value = .295 and .367 respectively). Table 2 shows that 76.47% of the 102 patients who responded to treatment had the TT genotype, 20.59% had the TC genotype and 2.94% had the CC genotype. The TT genotype was found in 60.2% of people who did not respond to treatment. About 32.65% and 7.14% of non‐responders had TC and CC genotypes respectively. In three models: dominant, recessive and co‐dominant, the variant investigated in the dominant model declines the HbA1C levels. In fact, as compared to the T/C + C/C genotype, the T/T genotype improves reducing HbA1C (OR = 2.48, CI = 1.17–3.99, *p*‐value = .014). In addition, the rs2250486 T allele was found to be more effective in reducing HbA1C levels (OR = 2.01, CI = 1.2–3.42, *p*‐value = .008).

**TABLE 2:** Genotypes and allele frequencies of rs2250486 and rs67238751 in responder and non‐responder groups.

Variant	Model	Genotype	Responder	Non‐responder	OR (95%Cl)	*p*‐value
rs2250486	Dominant	T/T T/C+C/C	78 (76.47%) 24 (23.53%)	59 (60.2%) 39 (39.8%)	Ref 2.14 (1.3‐17.99)	.014
	Co‐Dominant	T/T+C/C T/C	81 (79.41%) 21 (20.59%)	66 (67.35%) 32 (32.65%)	Ref 1.87 (0.3‐99.58)	.0549
	Recessive	T/T+T/C C/C	99 (97.06%) 3 (2.94%)	91 (92.86%) 7 (7.14%)	Ref 2.53 (0.12‐68.0)	.1865
	Allelic	T C	177 (86.76%) 27 (13.24%)	150 (76.53%) 46 (23.47%)	2.01 (1.3‐2.42)	.008
rs67238751	Dominant	C/C C/T+T/T	76 (74.51%) 26 (25.49%)	69 (70.41%) 29 (29.59%)	Ref 1.22 (0.2‐66.29)	.5164
	Co‐Dominant	C/C + T/T C/T	78 (76.47%) 24 (23.53%)	71 (72.45%) 27 (27.55%)	Ref 1.23 (0.2‐65.34)	.5145
	Recessive	C/C + C/T T/T	100 (98.04%) 2 (1.96%)	96 (97.96%) 2 (2.04%)	Ref 1.04 (0.8‐12.82)	.9678
	Allelic	C T	176 (27.76%) 28 (13.73%)	165 (84.18%) 31 (15.82%)	1.18 (0.2‐67.06)	.5558

#### 3.2.2 rs67238751 (C > T)

According to Table 2, out of 102 patients who responded to treatment, 74.51% had CC genotype, 23.53% had CT genotype, and 1.96% had TT genotype. Furthermore, in the non‐responders, 70.41% CC, 27.55% CT and 2.04% had TT genotype. The responder and non‐responder groups showed no deviations from Hardy–Weinberg equilibrium based on the data analysis (*p*‐value = .947 and .731 respectively). The frequency of genotypes of rs67238751 C > T in three models: dominant, recessive, and co‐dominant, as well as the abundance of alleles, are shown in Table 2. The variant tested did not demonstrate any significant relationship with the decrease in HbA1C levels after 6 months of metformin treatment in any models.

### 3.3 Comparison of anthropometric and biochemical parameters in variant rs2250486

Table 3 shows the changes in biochemical and anthropometric parameters in the responder and non‐responder groups according to T/T genotype and C allele carriers of rs2250486, after 6 months of metformin monotherapy. In the responder group, the BMI parameter in both T/T genotype and C allele carriers was not significantly reduced (*p*‐value = .541 and .5144 respectively). After 6 months of treatment, SBP declined significantly in both groups, although it was even lower in individuals with the T/T genotype (*p*‐value = .0001). In T/T genotype responders, the DBP parameter fell significantly (*p*‐value = .0004), but not in C allele participants (*p*‐value = .3144). After 6 months of metformin treatment, serum levels of TC, HbA1C and FBS dropped significantly in both groups (*p*‐value = <.0001, <.0001, <.0001 respectively). In addition, the average HDL‐C in both the C allele carriers and T/T genotype groups was not substantially lower than before the treatment (*p*‐value = .3065 and .576 respectively). The C allele carriers group had a substantial reduction in TG parameter (*p*‐value = .0077). However, the T/T genotype group had no significant drop (*p*‐value = .2787). And finally, the LDL‐C parameter in T/T genotype group demonstrates a significant decrease (*P*‐value = .0229). sdLDL showed a significant decrease in T/T and T/C + C/C genotypes only in responder group (*p*‐value = .0423, .0067 respectively). However, in the non‐responder groups, Individuals with the T/T genotype did not exhibit a significant reduction in the investigated parameters after 6 months of metformin treatment except for HbA1C levels that dropped considerably (*p‐*value = .007). Only DBP and HbA1C levels were significantly lower in C allele carriers (*p*‐value = .0279 and .010 respectively). However, in general the drop in HbA1C in these patients was insufficient to put them in the responder group (reduction was <1%).

**TABLE 3:** Comparison of anthropometric and biochemical parameters before and after treatment in T/T and T/C + C/C groups in Responder and non‐responder groups according to the genotypes of rs2250486.

Parameters	Responders						Non‐responders					
	T/T			T/C + C/C			T/T			T/C + C/C		
	Baseline	After 6 months	*p*‐value	Baseline	After 6 months	*p*‐value	Baseline	After 6 months	*p*‐value	Baseline	After 6 months	*p*‐value
BMI (kg/m^2^)	30.35 ± 4.57	29.92 ± 4.4	.5471	31.09 ± 5.23	30.11 ± 5.1	.5144	30.88 ± 3.9	30.67 ± 3.77	.7667	31.19 ± 5.32	30.94 ± 5.01	.8339
SBP (mm Hg)	131.09 ± 8.08	124.74 ± 6.93	.0001	130.83 ± 8.1	123.96 ± 7.66	.0042	130.08 ± 8.12	127.2 ± 9.25	.0748	128.46 ± 8.2	126.15 ± 8.31	.2209
DBP (mm Hg)	76.6 ± 4.73	73.85 ± 4.76	.0004	75.21 ± 5	73.75 ± 4.95	.3148	74.58 ± 4.85	75.93 ± 5.04	.1392	75.64 ± 5.4	73.08 ± 4.68	.0279
TG (mg/dL)	150.76 ± 14.86	147.83 ± 18.52	.2787	152.17 ± 16.4	137.92 ± 18.8	.0077	147.17 ± 16.17	146.95 ± 16.2	.9413	145.72 ± 15.0	148.56 ± 16	.4203
LDL‐C (mg/dL)	98.32 ± 14.42	93.41 ± 12.17	.0229	99.46 ± 14.7	95.17 ± 13.88	.3044	97.88 ± 13.74	97 ± 13.84	.7291	97.67 ± 13.79	96.33 ± 13.93	.6722
HDL‐C (mg/dL)	35.47 ± 3.81	36.67 ± 3.97	.0576	36.92 ± 3.41	38 ± 3.83	.3065	35.39 ± 3.94	35.78 ± 4.87	.6337	35.72 ± 4.05	35.72 ± 4.38	.0000
sdLDL‐C (mg/dL)	4.29 ± 0.56	4.08 ± 0.7	.0423	4.14 ± 0.47	3.67 ± 0.67	.0067	4.21 ± 0.67	4.19 ± 0.77	.8776	4.13 ± 0.64	4.21 ± 0.66	.5847
TC (mg/dL)	167.79 ± 18.07	151.01 ± 18.87	<.0001	175.38 ± 16.4	149.08 ± 17.2	<.0001	166.83 ± 15.93	167.15 ± 18.3	.9190	164.13 ± 16.5	164.51 ± 18.0	.9221
FBS (mg/dL)	166.64 ± 18.22	137.26 ± 16.96	<.0001	168.42 ± 21.3	134.5 ± 10.1	<.0001	164.27 ± 17.86	162.24 ± 18.8	.548	161.44 ± 20.9	159.85 ± 20.8	.738
HbA1c (%)	7.44 ± 0.38	6.07 ± 0.25	<.0001	7.45 ± 0.36	6.09 ± 0.27	<.0001	7.35 ± 0.47	7.03 ± 0.53	.0007	7.34 ± 0.5	7.03 ± 0.55	.0110

### 3.4 Comparison of anthropometric and biochemical parameters in variant rs67238751

Table 4 represents a comparison of the examined parameters in the responder and non‐responder groups with the T allele carriers and the C/C genotype before and after 6 months of metformin monotherapy. The mean BMI, HDL‐C and TG parameters with the C/C genotype in responder group did not appear to be reduced in this table (*p*‐value = .4349, .1202 and .1047 respectively). The SBP parameter drops in both T allele carriers (*p*‐value = .0128) and C/C genotype, but the latter group's decline is considerably more substantial (*p*‐value = <.0001). The DBP parameter was considerably lower in people with the C/C genotype (*p*‐value = .0015), but not in people with the T allele carriers (*p*‐value = .0956). The T allele carriers have significantly lower mean TC, FBS and HbA1C values (*p*‐value = .006, <.0001 and <.0001 respectively). In the C/C genotype group, this drop is likewise significant (*p*‐value = <.0001, <.0001 and <.0001 respectively). It is worth to mention that, sdLDL parameters in C/C and C/T + T/T in responder group had a meaningful reduction (*p*‐value = .0273, .272 respectively). Finally, in the C/C genotype group, the LDL‐C parameter was considerably reduced (*p*‐value = .0356), whereas this was not detected in the T allele group (*p*‐value =.1834). As shown in Table 4, in the non‐responder group with the T allele carriers and the C/C genotype, except for the HbA1C (*p*‐value = .0001), there was no significant reduction in any of the parameters before and after metformin treatment. Moreover, the reduction in HbA1C levels in these people was not significant enough to put them in the responder group (reduction was <1%). Table 5 reveals that rs67238751 and groups (responders & non‐responders) have interaction on various biochemical and anthropometric parameters. According to Table 5, as expected, there is a significant interaction between rs67238751 and groups on HbA1c and FBS. (*p*‐value = <.0001, <.0001 respectively). Additionally, the groups interaction on TC is meaningful either way (*p*‐value = <.0001). Despite the effect of groups on SBP, there was no evidence of interaction (*p*‐value = .12764).

**TABLE 4:** Comparison of anthropometric and biochemical parameters before and after treatment in C/C and C/T + T/T groups in Responder and non‐responder groups according to the genotypes of rs67238751.

Parameters	Responders						Non‐responders					
	C/C			C/T + T/T			C/C			C/T + T/T		
	Baseline	After 6 months	*p*‐value	Baseline	After 6 months	*p*‐value	Baseline	After 6 months	*p*‐value	Baseline	After 6 months	*p*‐value
BMI (kg/m^2^)	30.91 ± 4.58	30.33 ± 4.47	.4349	29.42 ± 5.02	28.9 ± 4.67	.6990	30.99 ± 4.1	30.79 ± 3.92	.7668	31.03 ± 5.39	30.76 ± 5.13	.8426
SBP (mm Hg)	131.58 ± 7.62	124.87 ± 7.21	<.0001	129.42 ± 9.2	123.65 ± 6.72	.0128	129.2 ± 8.16	126.59 ± 9.09	.0784	130 ± 8.24	127.24 ± 8.41	.2121
DBP (mm Hg)	76.58 ± 4.71	74.08 ± 4.81	.0015	75.38 ± 5.08	73.08 ± 4.71	.0956	74.71 ± 4.92	75.29 ± 5.14	.4994	75.69 ± 5.46	73.62 ± 4.8	.1312
TG (mg/dL)	151.43 ± 15.44	146.95 ± 18.57	.1074	150.08 ± 14.6	141.27 ± 19.9	.0751	146.42 ± 14.66	147.7 ± 16.28	.6295	147 ± 18.07	147.34 ± 15.9	.9389
LDL‐C (mg/dL)	99.04 ± 14.45	94.33 ± 12.89	.0356	97.27 ± 14.57	92.35 ± 11.58	.1834	96.77 ± 13.77	96.41 ± 13.77	.8774	100.24 ± 13.4	97.52 ± 14.11	.4540
HDL‐C (mg/dL)	35.78 ± 3.94	36.79 ± 4.05	.1202	35.92 ± 3.2	37.54 ± 3.72	.0996	35.94 ± 3.94	35.84 ± 4.61	.8896	34.52 ± 3.92	35.55 ± 4.86	.3758
sdLDL‐C (mg/dL)	4.27 ± 0.57	4.04 ± 0.7	.0273	4.2 ± 0.46	3.81 ± 0.73	.0272	4.13 ± 0.65	4.19 ± 0.7	.598	4.31 ± 0.67	4.23 ± 0.8	.6947
TC (mg/dL)	169.47 ± 17.75	150.5 ± 18.32	<.0001	169.88 ± 18.7	150.73 ± 19.1	.0006	166.77 ± 16.08	167.38 ± 19.3	.8411	163.34 ± 16.3	163.07 ± 14.7	.9464
FBS (mg/dL)	166.67 ± 18.63	136.29 ± 16.5	<.0001	168.19 ± 20.0	137.54 ± 12.9	<.0001	163.55 ± 18.49	161.01 ± 19.9	.4401	162.17 ± 20.8	161.93 ± 18.9	.9636
HbA1c (%)	7.46 ± 0.36	6.07 ± 0.23	<.0001	7.39 ± 0.4	6.1 ± 0.32	<.0001	7.33 ± 0.48	6.99 ± 0.51	.0001	7.37 ± 0.48	7.13 ± 0.58	.0891

**TABLE 5:** The interaction of rs67238751 and groups on biochemical and anthropometric parameters.

Parameters	*p*‐value		
	rs67238751	Groups (responders & non‐responders)	Interaction
BMI	.10922	.38035	.98614
SBP	.53830	<.0001	.12764
DBP	.52890	.00642	.04420
TG	.51928	.15570	.20284
LDL	.47944	.03143	.54372
HDL	.13037	.08407	.51620
sdLDL	.05102	.5239	.8692
TC	.00541	<.0001	<.0001
FBS	<.0001	<.0001	<.0001
HbA1c	<.0001	<.0001	<.0001

## 4 DISCUSSION

Type 2 diabetes is a metabolic disease that manifests as a chronic disease characterized by elevated blood glucose concentration. Because it raises the risk of heart disease, stroke, peripheral neuropathy, renal disease and amputation, and is linked to a shorter life expectancy.^15^ Diabetes has quadrupled in global prevalence during the previous three decades, and it is now the ninth greatest cause of mortality. Asia is a significant hotspot for the global T2DM epidemic, which is quickly growing.^16^ The most frequent treatment for T2DM is metformin, which reduces blood glucose concentrations in these patients. In recent years, studies have found that this medication has a remarkable effect on cardiovascular disease, neurological ailments and polycystic ovarian syndrome.^17^

According to clinical trials, more than a third of those taking metformin as a single dose do not achieve low fasting glucose levels. Changes in genes involved in the pharmacokinetics and pharmacodynamics of metformin could be the major reason for these people's lack of responsiveness to this medication.^18^ Many genetic variations are discovered using eQTL analysis. The availability of eQTL data from tissues linked to glycemic homeostasis in humans could help researchers better understand the metabolic processes that lead to T2DM.^19^ MATE1 receptor on renal tubular cells, together with the MATE2 receptor, is responsible for the excretion of metformin in the urine.

Studies in healthy volunteers and cell lines have shown that some of these genes can affect the distribution and elimination of metformin.^20^ In this regard, due to the high prevalence of T2DM on the one hand and its possible relationship with the variants of the *SLC47A1* gene with the use of metformin, on the other hand, we decided to assess the association of rs2250486 and rs67238751 intronic variants in the *SLC47A1* gene with response to metformin for the first time in the Iranian population with T2DM. To our knowledge, this is the first study to investigate the impact of these variants on metformin therapy response, and no previous research has been done on the rs2250486 and rs67238751 variants. The reason for the interest in studying two intronic variants is the studies conducted on these types of variations. Introns contain functional polymorphisms that may have an impact on how genes are expressed. Some of these intronic variations might possibly increase disease risk or alter the connection between genotype and phenotype. However, some intronic polymorphic variants can still affect the splicing phenotype by being found within an intron splice enhancer or branchpoint site, or by activating a cryptic splice site, even though they do not reside within the splice junctions. Such variants have probably been significantly under‐assessed up to this point. Many of these variations are single nucleotide variations, although others may also involve insertions or deletions. From a biological perspective, this is a more interesting subset of intronic variations to research because the processes by which these variants affect splicing are frequently obscure and sometimes rather subtle.^21^

In this research, patients who used metformin only in the first 6 months of treatment after diagnosis were studied. It should be noted that the average dose of metformin in this study was 1000 mg/day. In the present study, we observed a significant association between the rs2250486 variant of the *SLC47A1* gene and its efficacy on reducing HbA1C concentrations in these patients. According to our findings, patients who responded satisfactorily to treatment after 6 months had a greater frequency of allele T of the rs2250486 variant, and T/T genotype were significantly related to control T2DM. Metformin has also been demonstrated to have a positive impact on lipid profiles which is consistent with the fact that metformin, in addition to its antidiabetic action, also plays a role in lipid peroxidation. It should be emphasized that; 23 individuals in the responder group and 18 patients in the non‐responder group were receiving statin medication. Our results showed that T/C + C/C and T/T genotypes were substantially related to managed T2DM, and the frequency of allele T of the rs2250486 variant was higher in individuals who responded well to therapy after 6 months. Data from the GTEx portal suggests that T allele carriers may have a higher expression of the MATE1 protein in the liver, which would improve metformin's effect on the organ. After 6 months of metformin monotherapy, in the responder group of rs2250486 variant, SBP, DBP, LDL, TC, FBS, sdLDL and HbA1C were considerably reduced in those with T/T genotype and with C allele carriers, SBP, TG, TC, FBS, sdLDL and HbA1C were decreased significantly. Although there was no significant relationship between the rs67238751 variant and decreasing HbA1C levels, various anthropometric and biochemical parameters in the responder group were correlated with this variant. SBP, DBP, TC, LDL, FBS, sdLDL and HbA1C values were shown to be significantly lower in individuals with the C/C genotype. This drop was also seen in SBP, TC, FBS, sdLDL and HbA1C values in people who had the T allele carriers. Due to one of our hypothesis and the results from Table 5, with the interaction of this variant and groups (responder & non‐responder) on some of these parameters, we cannot discount the possibility that some of these biochemical and anthropometric parameters are correlated with the rs67238751. According to a study conducted on 800 individuals with T2DM in 2018, rs77474263 and rs77630697 genetic variants of the *SLC47A1* gene were found to be an essential determinant in metformin therapy response in Pakistani patients. There was also a correlation between some of the biochemical and anthropometric parameters with these variants.^22^ On the other hand, the number of studies on the same variants of the *SLC47A1* gene, have not shown similar results. In 2012, a study was performed on 148 patients with T2DM for *SLC47A1*, rs2289669. They discovered that those with the A‐allele carriers had a twofold lower HbA1c levels than those with the G‐allele carriers.^20^ However, in another study of the same variant, rs2289669 was discovered to have no influence on metformin efficacy.^23^ One of the controversial explanations could be that different nations have different alleles, and another reason could be the sample size, which can affect the accuracy of the results.

The results of this study may be effective in the treatment of patients with T2DM. However, the small sample size of the present study can be considered a limitation of this study. We hypothesize that the variant is related to metformin clearance, based on known functions of the gene, but without the serum concentration of metformin or medication adherence data there is no direct evidence that the changes in HbA1C are directly related to difference in metformin clearance. Therefore, further research in different study groups and wider sample sizes is needed to elucidate the role of the two variants rs2250486 and rs67238751 in the *SLC47A1* gene with an effective response to metformin.

The human genome has several genetic variations that have been found. Single nucleotide variant's functional impacts have been carefully examined. However, due to their complexity or a lack of evidence, the combined effects of many variations in the same genes have been largely disregarded. Due to present technological limitations, it is still difficult to deduce the genotypes of genes, and there is a lack of data that can be used to analyse the impact of different variations on the same gene. Future research on the combined efficacy of variations and responsiveness to metformin is hoped to be conducted.

Due to financial constraints, serum levels of insulin and metformin were not measured and an mRNA‐based study was not performed. It is hoped that further studies on different variants of the *SLC47A1* gene will provide more information on the efficacy of metformin in T2DM patients.

## 5 CONCLUSION

It seems that the rs2250486 variant might be an important factor in reducing HbA1C concentration in T2DM patients in the Iranian population, and in contrast to rs67238751 in response to metformin, no relationship was found. However, a larger sample size is required to complete this result.

## AUTHOR CONTRIBUTIONS

**Armina Semnani:** Conceptualization (equal); data curation (equal); formal analysis (equal); methodology (equal); writing – original draft (lead). **Faranak Kazerouni:** Data curation (equal); formal analysis (equal); validation (equal); writing – review and editing (equal). **saeid kalbasi:** Data curation (equal); formal analysis (equal); validation (equal). **Zahra shahrokhi:** Data curation (supporting); formal analysis (equal); validation (equal). **Ali Rahimipour:** Conceptualization (equal); funding acquisition (equal); project administration (lead); supervision (lead).

## FUNDING INFORMATION

The present article is financially supported by ‘Research department of the school of Medicine‐Shahid Beheshti University of Medical Sciences’ (Grant No: 24139).

## CONFLICT OF INTEREST STATEMENT

The authors have no relevant financial or non‐financial interests to disclose.

## ETHICAL APPROVAL

This study is performed in line with the principles of the Declarations of Helsinki. Approval was granted by the ethics committee of Shahid Beheshti University of Medical Sciences (No: IR.SBMU.MSP.REC.1399.119).

## CONSENT TO PARTICIPATE

Informed consent was obtained from all individual participants included in the study.

## CONSENT FOR PUBLICATION

Not applicable.

## Data Availability

Data will be made available on reasonable request.
